# A CDK-Dependent Phosphorylation of a Novel Domain of Rif1 Regulates its Function during Telomere Damage and Other Types of Stress

**DOI:** 10.1080/10985549.2023.2193768

**Published:** 2023-05-04

**Authors:** Cameron M. Robertson, Yuan Xue, Shobir Chowdhury, Laura Maringele

**Affiliations:** Newcastle University Biosciences Institute, Newcastle University, Newcastle upon Tyne, UK

**Keywords:** Rif1, telomere, budding yeast, DNA damage checkpoints, cyclin-dependent kinases

## Abstract

Rif1 mediates telomere length, DNA replication, and DNA damage responses in budding yeast. Previous work identified several posttranslational modifications of Rif1, however none of these was shown to mediate the molecular or cellular responses to DNA damage, including telomere damage. We searched for such modifications using immunoblotting methods and the *cdc13-1* and *tlc1Δ* models of telomere damage. We found that Rif1 is phosphorylated during telomere damage, and that serines 57 and 110 within a novel phospho-gate domain (PGD) of Rif1 are important for this modification, in *cdc13-1* cells. The phosphorylation of Rif1 appeared to inhibit its accumulation on damaged chromosomes and the proliferation of cells with telomere damage. Moreover, we found that checkpoint kinases were upstream of this Rif1 phosphorylation and that the Cdk1 activity was essential for maintaining it. Apart from telomere damage, S57 and S110 were essential for Rif1 phosphorylation during the treatment of cells with genotoxic agents or during mitotic stress. We propose a speculative “Pliers” model to explain the role of the PGD phosphorylation during telomere and other types of damage.

## INTRODUCTION

Telomeres are dynamic structures at the ends of eukaryotic chromosomes, composed of guanine-rich repetitive DNA sequences and associated proteins. These structures are difficult to maintain, especially in mammalian cells. One reason is that somatic cells lack telomerase activity, the enzyme required to counteract the loss of telomeric DNA during DNA replication.^1^ Another reason is that telomeres accumulate oxidative damage, due to their high guanine content and decreased DNA repair.^2–4^ Moreover, DNA repair enzymes like Exo1 enhance the damage, by degrading telomeric DNA.^5^^,^^6^ The main consequence of damaging the telomere is telomere shortening, which has been correlated with age-associated, degenerative diseases, immune deficiency and bone marrow failure.^7^

The amount of telomere damage acts as a threshold for different cellular responses. A small amount of telomere damage can be tolerated in yeast, with help from the Rif1 protein.^8^^,^^9^ Above this threshold, the DNA damage checkpoints pathways detect the dysfunctional telomeres and trigger a transient cell cycle arrest. This arrest could become permanent if the damage cannot be repaired, in which case it is referred to as cellular or replicative senescence. Cellular senescence plays numerous roles in cancer, implicated both in the development of malign lesions and the protection against tumorigenesis.^10^^,^^11^

To understand the cellular responses to telomere damage, we focused on Rif1 protein. Rif1 is well conserved from yeast to man, with multiple functions in telomere homeostasis, DNA damage repair, and DNA replication. As we mentioned above, budding yeast Rif1 affects the threshold of cellular responses to telomere damage.^8^^,^^9^ Another function of Rif1 is to regulate telomerase access to telomeres and therefore, telomere length.^12^ In mammalian cells, Rif1 is recruited to double strand breaks (DSBs) by 53BP1 (homologue of yeast Rad9) to suppress the 5′ end resection and divert their repair, from a BRCA1-mediated homologous recombination, to nonhomologous end joining.^13–18^ Rif1 also appears to promote NHEJ in yeast^19^ through yet unknown mechanisms. The role of Rif1 in DNA replication is to block the origin firing, by recruiting protein phosphatase 1 (PP1) to de-phosphorylate Mcm4 in the prereplication complex.^20–23^

Several domains of Rif1 have been identified: in budding yeast, the N-terminus domain (NTD) has been cocrystalized with DNA (aa 177–1283) and is considered relevant for the association of Rif1 with DNA.^19^ The PP1 binding domain has two canonical PP1 binding motifs, RVxF and SILK, located at the N-terminus in yeast (aa 114–320). The C-terminus is relevant for DNA replication in mammals, as is the DBf4 binding domain, aa 1790–1916.(24, S 32).^24^^,^^25^ The Rap1 binding motif (RBM) at the C-terminus is important to localize Rif1 to native telomere in yeast (26). The HEAT repeats (aa 177–996), particularly aa 436–577, have been recently identified as important for telomere length regulation in yeast.^26^

Several post-translational modifications of Rif1 have been also identified. S-acylation of Rif1 at C466 and C473 appears to promote NHEJ in budding yeast.^26^ Ubiquitination and SUMOylation are required to dissociate 53BP1-Rif1 from the DSB in mammalian cells.^26^^,^^27^ Phosphorylation of Rif1 by CDK and DDK is relevant for the suppression of late firing origins of replication.^20^^,^^28^

Since known posttranslational modifications of Rif1 do not explain its role in the cellular responses to telomere damage, we searched for new modifications, using Western blotting. We report that yeast Rif1 is phosphorylated and that the S57, S110 residues in a domain that we now call the phospho-gate domain (PGD) are important for it. We propose that PGD acts to regulate the association of Rif1 with damaged subtelomeres and other chromosomal regions and suggest a speculative model to explain this function. The “Pliers” model proposes that Rif1 dimers assemble onto DNA as a pair of “molecular pliers”, with the PGD at the tip. The pliers are “opened” by phosphorylation, and so the association of Rif1 with DNA becomes unstable. This model could be relevant for other types of cellular damage, since we found that Rif1 is phosphorylated and that S57, S110 are important during several other genotoxic stresses, and even during mitotic spindle poisoning with nocodazole.

## RESULTS

### Rif1 is phosphorylated during telomere damage

To better understand how Rif1 operates during telomere damage, we searched for new posttranslational modifications of Rif1. To induce telomere damage, we used the temperature sensitive *cdc13-1* yeast cells. These cells have functioning telomeres at low temperature. When incubated at restrictive temperature however, telomeres and adjacent chromosomal regions become resected by Exo1 and other nucleases. Single-stranded DNA (ssDNA) progressively accumulates in these regions, and triggers a checkpoint response, arresting the cell cycle in the G2/M phase. Rif1 does not directly regulate resection during the telomere damage, however it regulates the checkpoint response by limiting the association of checkpoint proteins with damaged regions through an anticheckpoint function.^9^

**FIG 1:**
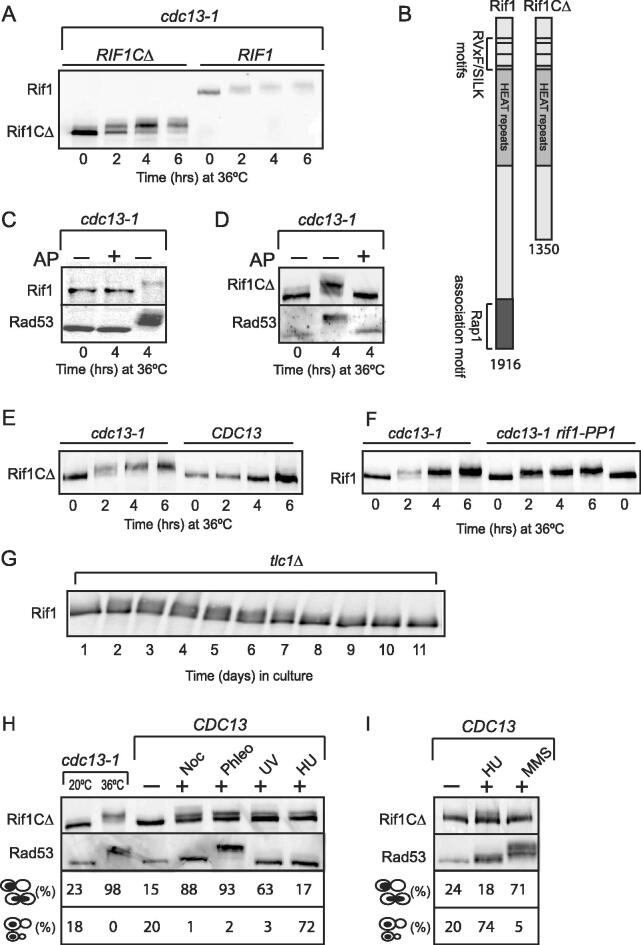
The phosphorylation of Rif1. (A) WB showing the migrating forms of Rif1CΔ (left half) and Rif1 (right half) present in *cdc13-1* cell cultures during a time course of 6 h at 36 °C. (B) A schematic representation of Rif1 and Rif1CΔ showing the approximate location of the main motifs. (C) WB of Rif1 (top) and Rad53 (bottom) from *cdc13-1* cultures incubated at either permissive temperature (time 0) or for 4 h at 36 °C. The extracts at 36 °C have been treated with AP (+) or mock treated (−). (D) The same as in C, except that we detected Rif1CΔ instead of Rif1. (E) WB of Rif1CΔ in *cdc13-1* (left half) or *CDC13* (right half) during a 6 h time course at 36 °C. (F) WB of Rif1 (left half) and Rif1-PP1 (right half) in *cdc13-1* cultures during a 6 h time course at 36 °C. (G) Cells from a *tlc1Δ RIF1-MYC* strain, taken directly from the germination plate were grown in liquid culture for 11 days. Cells were diluted every 24 h to 1 × 10^5^ cells/mL and samples collected for immunoblotting with Myc antibody. (H) WB of Rif1CΔ and Rad53 in *cdc13-1* cultures (first two columns) and *CDC13* cultures (the next five columns) treated as follows (from left to right): 20 °C; 36 °C; mock; nocodazole; phleomycin; ultraviolet light; hydroxyurea. The numbers shown below each WB column are the percentage of large budded (top) or small budded (bottom) cells in the respective cell culture. (I) Same as in H, except that only *CDC13* cells were treated, as follows: mock; hydroxyurea; methyl methane sulfonate.

To detect Rif1, we used an epitope tagged version, as well as a truncated form, called Rif1CΔ. Rif1CΔ lacks the C terminus, also known as the terminal 1351–1916 amino acids (Fig. 1B) however it still retains the anticheckpoint function, as previously described.^9^ Overnight cultures of *cdc13-1 RIF1-MYC* and *cdc13-1 RIF1CΔ -MYC* cells, grown at 20 °C, were diluted and shifted to restrictive temperature (36 °C) with samples being collected every second hour. We used Western blotting (WB) to detect Rif1 in these samples (Fig. 1A). A relatively sharp protein band was observed at time 0 (20 °C). This basic form appeared to be replaced by slower migrating forms at 36 °C (Fig. 1A). The interaction between the basic and the slower migrating forms was more evident for Rif1CΔ than Rif1, because sharper protein bands were obtained due to its lower MW.

By treating some of the protein extracts with alkaline phosphatase (AP) to remove potential phosphoryl groups, we found that the slower migrating forms of Rif1 and Rif1CΔ, detected after 4 h at 36 °C, were due to phosphorylation (Fig. 1C and 1D). Rad53, a checkpoint kinase known to be phosphorylated during telomere and other types of damage, was used as a positive control for this assay.

Since Rif1 phosphorylation was observed at 36 °C, we questioned whether the high temperature *per se* could have been responsible for this modification, independently of telomere damage. Therefore, we cultured *cdc13-1* and *CDC13* cells over 6 h at 36 °C, and compared the migration patterns of Rif1CΔ in these cultures. Phosphorylation of Rif1CΔ was observed in *cdc13-1*, but not in *CDC13* cells (Fig. 1E). This shows that Rif1 phosphorylation was dependent on telomere damage, rather than high temperature.

With further experiments, we tested whether the RVxF/SILK motifs of Rif1 were relevant for its phosphorylation. These motifs are important for the function of Rif1 in recruiting PP1 (protein phosphatase 1) during DNA replication.^29^^,^^30^ We found that Rif1 was still phosphorylated in cells with disrupted PP1 motifs (also known as *cdc13-1 rif1-PP1* cells) indicating that the RVxF/SILK motifs, and therefore PP1, were not important for Rif1 phosphorylation (Fig. 1F).

To determine whether Rif1 was phosphorylated during other types of telomere damage, we examined *tlc1Δ* cells lacking the *TLC1* gene, essential for telomerase function (Fig. 1G). In the absence of telomerase, telomeres progressively erode until cells enter a state of cell cycle arrest, called replicative senescence. In our experiments, *tlc1Δ RIF1-MYC* strains, taken directly from the germination plates, were inoculated in liquid YPD and diluted every day to 1 × 10^6^ cell/ml. Proliferation of *tlc1Δ RIF1-MYC* cells progressively declined during the first 7 days, followed by recovery due to the emergence of survivors, as previously found.^31^^,^^32^ WB analysis of protein extracts collected every 24 h showed that Rif1 became phosphorylated, and this modification appeared as early as day 2 and persisted until day 7, thus overlapping with the phase of growth decline known as replicative senescence (Fig. 1G).

### Rif1 is phosphorylated during several types of stress

To determine whether Rif1 was also phosphorylated during other types of stress, we treated wild-type cells (marked as *CDC13*) with the spindle poison nocodazole, or with genotoxic agents. Samples were collected after 3–4 h incubation with the indicated agents and analyzed by WB (Fig. 1H). Samples from the *cdc13-1* cells cultured at 20 °C or 36 °C were used as negative and positive controls for phosphorylation, respectively. To achieve a better separation between the basic and the slower migration forms by WB, we used Rif1CΔ. Excitingly, we observed the slower migration forms of Rif1CΔ in wild-type cells treated with nocodazole, phleomycin, UV radiation and hydroxyurea (Fig. 1H). The basic migration form was also present, suggesting that only a fraction of Rif1CΔ has been modified.

To understand the correlation between Rif1 phosphorylation and DNA damage responses, we analyzed the Rad53 phosphorylation and the cell cycle distribution. Rad53 is important for cell cycle arrest in response to DNA damage. We found that Rif1 phosphorylation appeared to loosely correlate with Rad53 phosphorylation, as well as with an accumulation of large budded or small budded cells, suggestive of cell cycle arrest in G2/M or S-phase, respectively (Fig. 1H). Interestingly, Rif1CΔ remained unphosphorylated in cells treated with the alkylating agent methyl methane sulfonate (MMS), despite a strong Rad53 phosphorylation and cell cycle arrest (Fig. 1I). Although we do not know the reason behind this behavior, we suggest that Rif1 could be directly affected by MMS in a way that prevents its phosphorylation.

In conclusion, Rif1 became phosphorylated during different types of stress, ranging from *cdc13-1*-induced telomere damage, to replicative senescence, spindle poisoning, UV radiation, replicative fork stalling and DNA breaks.

### S57 and S110 residues within a novel phospho-gate domain are important for Rif1 phosphorylation

**FIG 2:**
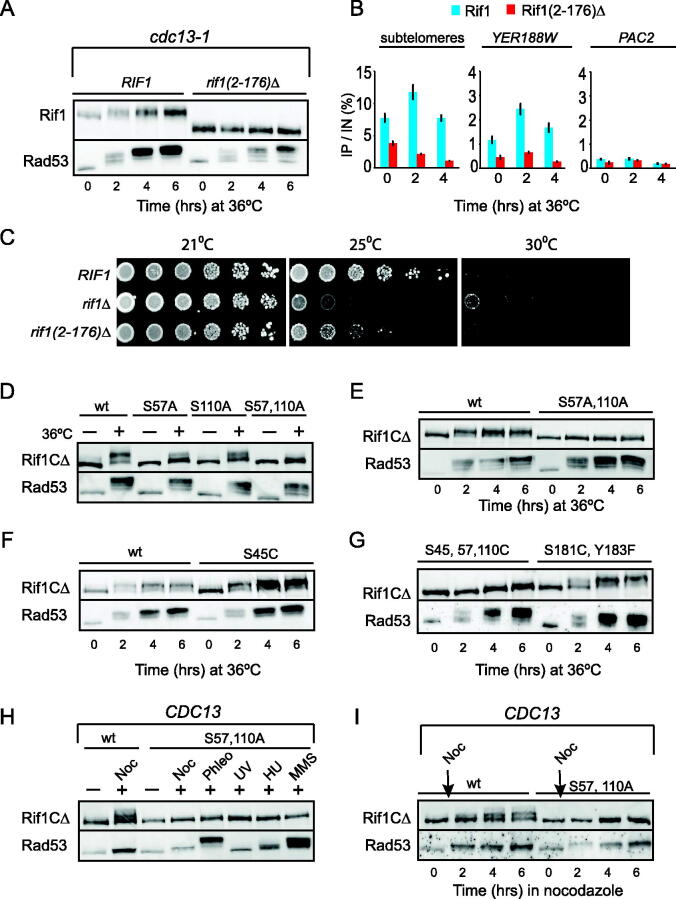
The importance of the residues 2-176, S57 and S110 for the phosphorylation of Rif1 and other events. (A) WB of Rif1 (top) and Rad53 (bottom) from *cdc13-1* cultures with full length Rif1 (left half) or Rif1(2-176)Δ (right half) during 6 h time course at 36 °C. (B) Chromatin immunoprecipitation showing the association of Rif1 (blue columns) or Rif1(2-176)Δ (red columns) with subtelomeres (left panel), *YER188W* (middle) or *PAC2* (right panel) in *cdc13-1* cultures, during 4 h time course at 36 °C. The columns represent the percentage of the immunoprecipitated DNA out of the input DNA (total DNA), quantified by qPCR using primers and Taqman probes specific to the locus/region indicated above each graph. (C) Growth of serially diluted droplets of *cdc13-1* cultures, with full length Rif1 (top), rif1Δ (middle) or rif1(2-176)Δ (bottom lanes) on plates incubated at different temperatures, indicated above each plate. (D) WB of Rif1CΔ and Rad53 in *cdc13-1* cultures after 4 h at either permissive (–) or restrictive, 36 °C, temperature (+). Native Rif1CΔ is marked as “wt” and is followed by Rif1CΔ with the following serine substitutions: S57A, S110A and S57,110A, as indicated above the columns. (E to G) WB of Rif1CΔ and Rad53 in *cdc13-1* cultures during 6 h experiments at 36 °C. The native form of Rif1CΔ is marked as “wt” (left half in 2E and 2 F). Mono (S45C), double (S57A, S110A) or triple (S45C, S57C, S110C) residue substitutions of Rif1CΔ, as well as the double residue substitution S181C, Y183F, are indicated above the respective halves of each subfigure. (H) WB of Rif1CΔ and Rad53 in *CDC13* cultures under stress. Rif1CΔ is either native (the first two columns, “wt”) or substituted at both S57 and S110 (the remaining columns). Cells were treated as follows, from left to right: mock; nocodazole; mock; nocodazole; phleomycin; UV light; hydroxyurea; MMS. (I) WB of Rif1CΔ and Rad53 in *CDC13* cultures during 6 h time course. Nocodazole was added to the cultures immediately after the “time 0” was collected. The native form of Rif1CΔ is on the left (wt); the double substituted form S57,110A is on the right.

To understand the location of Rif1 phosphorylation, we proceeded to map the relevant residues. We began by investigating a truncated form of Rif1 which lacked the residues 2–176. We found that this form of Rif1 showed no signs of phosphorylation by WB in *cdc13-1* cells exposed to 36 °C and marked as *rif1(2–176)Δ* in Fig. 2A, whereas the Rif1 full length protein and Rad53 were both phosphorylated (Fig. 2A). This suggests that residues 2–176 are important for phosphorylation.

To test the significance of these residues, we compared the ability of Rif1 and Rif1(2–176)Δ proteins to associate with chromosomes during telomere damage, using chromatin immunoprecipitation. The damage was induced by incubating the *cdc13-1* cultures for 4 h at 36 °C. We found that Rif1 significantly associated with subtelomeres and the adjacent *YER188W* locus, which are known for accumulating DNA damage in *cdc13-1* cells at 36°C, and very little with the much further *PAC2* locus (Fig. 2B) consistent with previous findings.^34^ In contrast, the association of Rif1(2–176)Δ with subtelomeres and *YER188W* was drastically reduced (Fig. 2B).

Rif1 is important for *cdc13-1* cells to grow at permissive temperature due to its anticheckpoint function.^9^ We asked whether Rif1 (2–176)Δ could still provide this function. We found that *rif1(2–176)Δ* cells grew poorly at the permissive temperature of 25 °C, although slightly better than *rif1Δ* cells, which failed to grow, as expected (Fig. 2C). We conclude that the first 176 amino acids are important for the function of Rif1, most likely due to the fact they are important for the phosphorylation and the association of Rif1 with chromosomes (Fig. 2A and 2B). We refer to the first 176 amino acids as the phospho-gate domain (PGD) of Rif1.

We used predictive software (KinasePhos) to map potentially phosphorylated residues within the first 176 amino acids of Rif1, and then substituted some of them with non-phosphorylable amino-acids like alanine or cysteine. We found that S57 and S110 were critical for Rif1CΔ phosphorylation during a *cdc13-1*-induced telomere damage (Fig. 2D). This was because the substitution of S57 or S110 with alanine has partially eliminated the slower migrating forms, with S57A having a stronger effect than S110A. Moreover, the combination of S57A and S110A in the same Rif1CΔ proteins completely eliminated the slower migrating forms otherwise detectable by WB. Rad53 phosphorylation, as a marker for DNA damage, was unaffected by these serine substitutions, suggesting that the DNA damage was also unaffected (Fig. 2D).

The importance of the S57 and S110 residues for Rif1 phosphorylation was further revealed during time-course experiments, in which protein extracts from *cdc13-1* cells were analyzed by WB at different time points during 6 h at 36 °C. In contrast to the pattern observed in Rif1CΔ lacking serine substitutions (marked as “wt” from “wild-type”), the combined S57A and S110A substitutions showed no phosphorylation at any collected time points (Fig. 2E). The Rad53 phosphorylation remained unaffected by S57A and S110A substitutions.

Other residues with a high score on KinasePhos were S45, S181 and Y183. These residues were analyzed during similar experiments to those described in Fig. 2E. It was clear that substitution S45C (Fig. 2F) or S181C, Y183F (Fig. 2G, right half) had little to no impact on Rif1CΔ phosphorylation detected by WB. In contrast, a combination of substitutions S45C, S57C and S110C (Fig. 2G, left half) eliminated the Rif1CΔ phosphorylation (also demonstrating that the effect was not limited to substitution with alanine). Rad53 phosphorylation appeared unaffected by any of the above mentioned substitutions.

An important question is whether residues S57 and S110 are also relevant for Rif1 phosphorylation during other types of stress. To address this question, we exposed wild-type cells with S57A and S110A substitutions in Rif1CΔ to the same stressors described in Fig. 2H and 2I. Excitingly, we found that S57 and S110 were also important for Rif1CΔ phosphorylation in response to nocodazole, phleomycin, UV or HU (Fig. 2H). A S57,110A combination also eliminated Rif1CΔ phosphorylation at any tested times during a 6 h course in nocodazole (Fig. 2I).

In conclusion, we have identified two residues (serine 57 and serine 110) important for Rif1 phosphorylation during telomere damage. We found that these residues are also important for Rif1phosphorylation during the treatment with nocodazole, phleomycin, UV radiation or hydroxyurea. These residues are within a novel phospho-gate domain (PGD).

### Cell cycle Arrest is important for Rif1 phosphorylation

**FIG 3:**
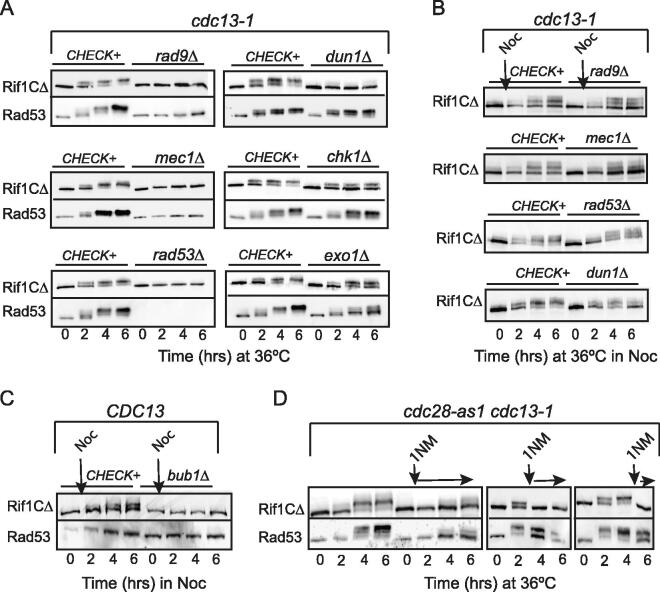
Kinases and other factors upstream of the Rif1 phosphorylation. (A) WB of Rif1CΔ and Rad53 in *cdc13-1* cultures during 6 h time course at 36 °C. The cell cultures are either checkpoint-proficient (the left half, marked as CHECK+ above the pictures) or have the following deletions of checkpoint or other genes: *rad9Δ*, *mec1Δ*, *rad53Δ*, *dun1Δ*, *chk1Δ*, *exo1Δ* (the right half; the respective deletion is indicated above the pictures). (B) WB of Rif1CΔ in *cdc13-1* cultures during 6 h time course at 36 °C. Nocodazole was added to each culture immediately after “time 0” (at permissive temperature) was collected. Additional mutations are indicated above the pictures. (C) as in B, except that cells are *CDC13* and the spindle checkpoint gene *BUB1* has been deleted. Rad53 WB is also shown. (D) WB of Rif1CΔ and Rad53 in *cdc28-as1 cdc13-1* cultures during 6 h time course at 36 °C. The initial culture, grown overnight at permissive temperature, has been split into four, each part being treated differently, as follows (from left to right): mock treated; 1-NM-PP1 added immediately after the “time 0” collection; 1-NM-PP1 added after 2 h at 36 °C; 1-NM-PP1 added after 4 h at 36 °C.

To identify the kinase(s) responsible for Rif1 phosphorylation, we analyzed the effect of DNA damage checkpoint kinases Mec1, Rad53, Chk1, Dun1, and that of Rad9, which is an upstream checkpoint protein, on the phosphorylation of Rif1CΔ, using *cdc13-1* cell cultures incubated for 6 h at 36 °C. Interestingly, we found that deletion of either *RAD9*, *MEC1*or *RAD53* genes from *cdc13-1* cells completely abolished Rif1CΔ phosphorylation (Fig. 3A). Phosphorylation of Rad53 was also abolished, as expected, since Rad9 and Mec1 are required for it. Deletion of *DUN1*, encoding a kinase downstream of Rad53, also abolished the majority of phosphorylated Rif1CΔ forms, whereas Chk1, acting in a parallel checkpoint pathway to Rad53,^35^ had significant, but lesser effects (Fig. 3A). Exo1, the exonuclease resecting chromosome ends in *cdc13-1* cells,^34^ also had an effect. These results placed Rad9, Mec1, Rad53, Dun1, and to a lesser extent Chk1 and Exo1, upstream of the Rif1 phosphorylation.

It is possible that some of these checkpoint kinases are directly phosphorylating Rif1. However, it is also possible that their effect is indirect, through affecting the cell cycle progression. To understand the effect of cell cycle arrest on Rif1 phosphorylation, we performed the same experiments as in Fig. 3A, except that we treated the *cdc13-1* cells with nocodazole, at the time we shifted the temperature to 36 °C. Treatment with nocodazole poisons the spindle, and therefore induces arrest at the anaphase-metaphase transition.^35^ Moreover, treatment with nocodazole also causes phosphorylation of Rif1 at residues S57 and S110 (Fig. 2G). Importantly, we found that upon addition of nocodazole to *cdc13-1* cultures and shift to restrictive temperatures, Rif1CΔ became phosphorylated, despite a *rad9Δ, mec1Δ*, *rad53Δ*, or *dun1Δ* mutation (Fig. 3B). A *bub1Δ* mutation, which abrogates the spindle checkpoint, thus permitting cells to escape arrest despite the presence of nocodazole,^36^ abrogated the Rif1CΔ phosphorylation induced by nocodazole in wild-type cells (Fig. 3C).

In conclusion, cell cycle arrest is very important for Rif1 phosphorylation during telomere damage. Several DNA damage checkpoint kinases and Exo1 are upstream of this phosphorylation event. However, if the cell cycle is arrested by other means than DNA damage (by spindle poisoning), then the DNA damage checkpoint proteins become redundant for Rif1 phosphorylation. Therefore, these proteins may have an indirect role in Rif1 phosphorylation, by arresting the cell cycle, although a more direct role in a fraction of cells cannot be excluded.

### Prolongated activity of Cdk1 is essential for Rif1 phosphorylation

To identify kinase(s) that may phosphorylate Rif1, we turned our attention to the cyclin-dependent kinase 1 (Cdk1). Cdk1 (Cdc28) is the main kinase regulating progression through the cell cycle in budding yeast, by associating with different cyclins and regulating a multitude of targets. Rif1 is a potential target of Cdk1 during DNA replication.^20^^,^^37^ Therefore, we hypothesized that Cdk1 could be upstream of the Rif1 phosphorylation occurring in response to telomere damage and other stresses. To investigate the effect of Cdk1, we used the allele *cdc28-as1.*^38^ This *CDK1* allele encodes a kinase with an enlarged ATP-binding pocket, which can be bound by a competitive inhibitor of ATP called 1-NM-PP1. Following incubation of cells in medium containing 1-NM-PP1, the kinase activity of Cdk1 is specifically inhibited, with the majority of cells accumulating in G2/M.^38^

To examine the effects of 1-NM-PP1 on Rif1CΔ phosphorylation, we cultured *cdc13-1 cdc28-as1* at 20 °C. Each culture was divided in two just before the temperature shift to 36 °C: one half received the inhibitor 1-NM-PP1, the other half was mock treated. Samples were taken every two hours and analyzed by WB for Rif1 and Rad53 phosphorylation (Fig. 3D). We detected a substantial decrease in the phosphorylated forms of Rif1CΔ in cells treated with the inhibitor, versus mock-treated cells. However, the inhibitor also caused a substantial decrease in Rad53 phosphorylation (Fig. 3D). This could be because Cdk1 kinase activity may be required for Rad53 phosphorylation in *cdc13-1* cells, similarly to what was shown in response to DNA damaging agents.^39–41^

To avoid the effect of Cdk1 on the Rad53, we allowed *cdc13-1 cdc28-as1* cells to undergo telomere damage, cell cycle arrest and Rad53 phosphorylation before being treated with the inhibitor. To do this, we divided this culture: to one half, we added 1-NM-PP1 immediately after collecting the 2 h sample; to the other half, after collecting the 4 h sample. Both cultures were then incubated for a total of 6 h at 36 °C. WB analysis of samples collected before adding the inhibitor showed that both Rad53 and Rif1CΔ became phosphorylated. Excitingly, addition of 1-NM-PP1 after 2 or 4 h at 36 °C completely eliminated this Rif1CΔ phosphorylation (Fig. 3D). Addition of 1-NM-PP1 after 2 or 4 h had a much lesser effect on Rad53 phosphorylation.

These results not only indicate that Cdk1 kinase is upstream of the Rif1 phosphorylation, but also that a prolonged activity of Cdk1 is required to maintain the Rif1 phosphorylation. This is because once the kinase function of Cdk1 is abolished, the phosphorylated forms of Rif1 are replaced by native forms of Rif1 on Western blots.

### Phosphorylation of Rif1 dependent upon S57, S110 inhibits proliferation of cells with telomere damage

**FIG 4:**
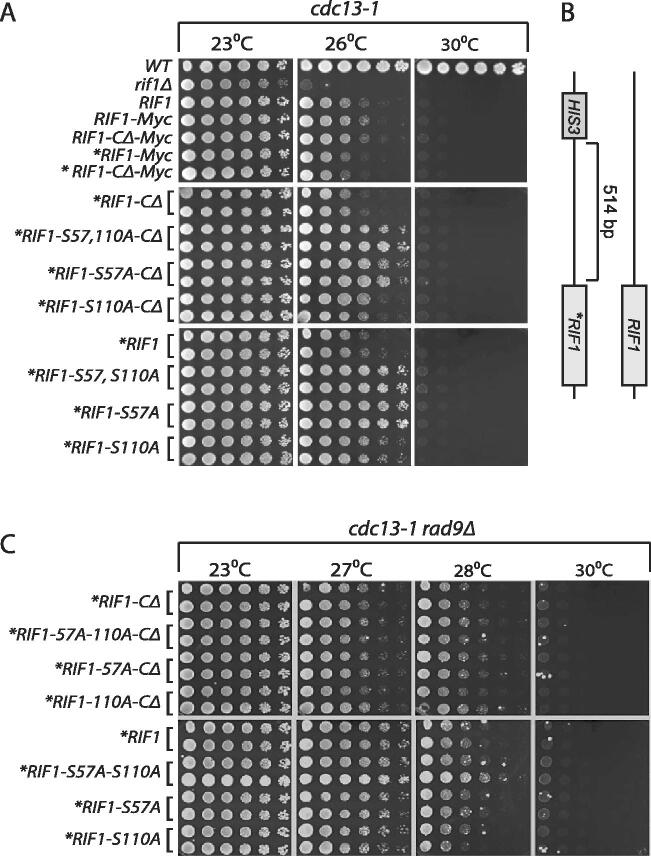
How different mutations in the *RIF1* gene or its promoter affected the cell fitness. (A) Growth of serially diluted droplets of *cdc13-1* cultures (except for the very first lane, “WT” which was an wild-type culture) with native Rif1 or mutated Rif1, on plates incubated at different temperatures, indicated above each plate. The asterisk indicates that a *HIS3* cassette has been inserted at 514 bp in front of the ATG of the *RIF1* gene. Two different isogenic strains are shown, except for the first plate. (B) Schematic representation of the *HIS3* cassette and the *RIF1* locus. (C) As in A, except that all cultures also have a *rad9Δ* mutation. Two different strains are shown for each genotype. The relevant incubation temperature has been increased compared to A, since it is known that a *rad9Δ* mutation increases the restrictive temperature of *cdc13-1*cells, from 26 °C to 27 °C.

Rif1 facilitates proliferation of *cdc13-1* cells with telomere damage, which translates into higher temperature tolerance than that of *rif1Δ cdc13-1* cells.^9^ To determine whether phosphorylation of Rif1 at S57 and S110 affected the temperature tolerance of *cdc13-1* cells, we performed spot assays, in which serially diluted droplets of cell culture were plated and incubated for a few days, at different temperatures (examples are shown in Fig. 4A).

Firstly, we tested whether the changes introduced in our mutants to permit selection of transformed cells and detection of Rif1 have affected the Rif1 function and thus, the temperature tolerance. These changes were the introduction of a *HIS3* cassette (*) upstream of the *RIF1* start codon and of a Myc tag at the C-terminus (Fig. 4B). We found that growth of strains and temperature tolerance were largely unaffected by the *HIS3* cassette and Myc tag (Fig. 4A).

Excitingly, all *cdc13-1* strains with relevant serine substitutions in Rif1 or Rif1CΔ grew better than unsubstituted homologues at the restrictive temperature of 26 °C. This effect was the strongest for S57A, S110A double substitution, closely followed by S57A, whereas the S110A substitution had smaller effects (Fig. 4A). Therefore, growth appeared to inversely correlate with degrees of Rif1 phosphorylation observed in Fig. 2D.

As further evidence that the growth advantage observed in S57A, S110A strains was due to Rif1 phosphorylation, we introduced an additional mutation in the genetic background, this time in the *RAD9* gene. We showed in Fig. 3A that a *rad9Δ* mutation abrogated the Rif1 phosphorylation. It is known that a *rad9Δ* mutation raises the restrictive temperature of *cdc13-1* cells to about 27–28 °C, by eliminating the cell cycle arrest.^42^ When we examined cell growth at these temperatures, we found that a *rad9Δ* mutation abrogated the growth advantage brought by the S57A and S110A substitutions in Rif1 or Rif1CΔ, since these strains showed similar growth to unsubstituted homologues at all tested temperatures (Fig. 4C). We concluded that S57 and S110 of Rif1 are moderating the proliferation of cells undergoing telomere damage, depending on their ability to become phosphorylated.

### Phosphorylation dependent upon S57, S110 inhibits the association of Rif1 with chromosomes during telomere damage

To understand the molecular effects of Rif1 phosphorylation, we asked whether S57 and S110, and therefore the Rif1 phosphorylation, were relevant for the association of Rif1 with chromosomes during telomere damage. The association of Rif1 with damaged chromosome regions is considered important for its molecular function.^19^ Therefore, we used chromatin immune-precipitation (ChIP) to detect Rif1 association with loci close to the telomere of chromosome V. A further locus, *PAC2,* was used as a control for unspecific DNA association. It is well documented that the telomere-proximal loci *YER190W*, *YER188W,* and *YER186C* accumulate DNA damage during *cdc13-1* experiments, whereas *PAC2* does not.^34^

**FIG 5:**
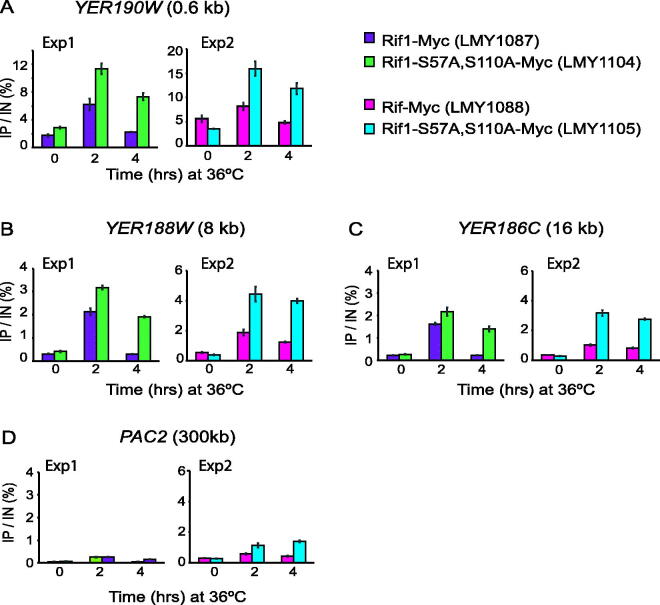
The association of the native Rif1 or serine substituted Rif1 with different loci. (A) The percentage of DNA immunoprecipitated with Myc antibodies, expressed from the total amount of DNA, quantified by qPCR in subtelomeres (at *YER190W*, more precisely). Data from two different, representative experiments (marked as Exp1 and Exp2) is shown in separate graphs, due to the slightly different levels of crosslinking and/or immunoprecipitation between experiments. The error bars represent the SD from three technical repeats. To obtain the data, different *cdc13-1* cultures were shifted from permissive (21 °C, time 0) to restrictive (36 °C) temperature and incubated for 4 h, to accumulate telomere damage. Samples were collected every second hour and then processed to obtain the percentage of immunoprecipitated DNA in strains with Rif1-myc (purple and red columns for Exp1 and Exp2, respectively) or Rif1-S57, S110A-myc (green and blue columns for Exp1 and Exp2, respectively). Different strains have been used: LMY1087 and LMY1088 are *cdc13-1 RIF1-MYC*; LMY1104 and LMY1105 are *cdc13-1 RIF1-S57A, S110A-MYC*. (B) As in A, except that the data is obtained at the *YER188W* locus, at circa 8 kb from the chromosome end. (C) data obtained at the *YER186C* locus, circa 16 kb from the chromosome end. (D) data obtained at the *PAC2* locus, circa 300 kb from the chromosome end.

Cultures of *cdc13-1 HIS3-RIF1-myc* and *cdc13-1 HIS3-RIF1*-*S57,110A-myc* strains were incubated at 36 °C for 4 h. Samples, collected every second hour, were analyzed by ChIP. At time 0 (under permissive temperature), both forms of Rif1 associated with *YER190W*, since normal subtelomeres contain TG repeats which are bound by Rif1 through Rap1. The small difference between the two Rif1 forms appeared to be strain specific (Fig. 5A). After 2 h at 36 °C, both Rif1 and Rif1-S57,110A accumulated at *YER190W*. However, the peak in the substituted form was almost 2-fold higher than that of Rif1. At 4 h, the difference in association between the two forms of Rif1 increased even further, with the peak in the substituted form up to 3-fold higher than that of Rif1 (Fig. 5A). This pattern was consistent between different strains and experiments (Exp1 and Exp2, Fig. 5A).

A similar pattern occurred at *YER188W* and *YER186C* loci, where the association of Rif1-S57,110A was about 2-fold higher (at 2 h) and up to 5-fold higher (at 4 h) than that of Rif1 (Fig. 5B, C). The larger difference at 4 h may have been caused by Rif1 disengaging from chromosomes (more obvious in Exp1), whereas the substituted form remained largely associated with chromosomal DNA. Little association was detected at the *PAC2* control locus (Fig. 5D).

In conclusion, the S57,110A form of Rif1 associated significantly more with chromosome regions than its unsubstituted homologue. Moreover, the S57,110A form appeared to have maintained its association with chromosome for at least 2 h longer than its homologue. Since S57 and S110 are responsible for Rif1 phosphorylation, these results suggest that Rif1 phosphorylation acts to limit and/or to remove Rif1 from chromosomes during telomere damage.

## DISCUSSION

**FIG 6:**
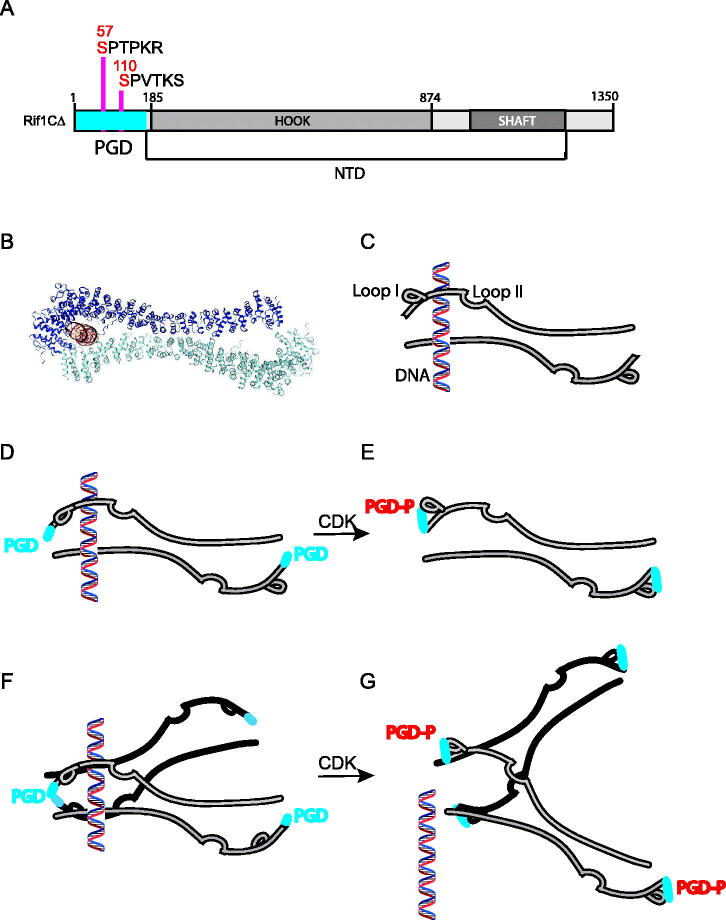
The Pliers model of Rif1 action. (A) Schematic representation of Rif1CΔ protein showing the approximative localization of the PGD and the NTD domains. (B and C) Schematic representation of the crystal structure of a Rif1 NTD dimer on DNA (B) with further detail (C), according to Longtine et al.^44^ (D) Same as in C, except the PGD has been added. (E) Same as in D, except that PGD is now phosphorylated and adopts an “open” configuration. (F) Schematic representation of two Rif1 NTD dimers as a molecular pliers, with PGD in “closed” configuration and DNA threaded through the cavity. (G) Same as in F, except that the PGD is now phosphorylated and the Rif1 pliers adopt an “open” configuration, thus releasing DNA.

We describe a new posttranslational modification of the DNA damage and replication protein Rif1. This modification is a S57 and S110 dependent phosphorylation, which occurred during telomere damage, UV radiation, spindle poisoning, hydroxyurea, and phleomycin treatments (Fig. 1 and 2) within a domain we called the phospho-gate domain (PGD, Fig. 6A). We have shown that Cdk1, several DNA damage checkpoint proteins and a spindle checkpoint protein were all upstream of the S57, S110 dependent, Rif1 phosphorylation (Fig. 3). Interestingly, the checkpoint proteins became redundant for Rif1 phosphorylation when the cell cycle was arrested chemically. Therefore, the role of the checkpoint proteins in RIF1 phosphorylation could be indirect, to induce cell cycle arrest and thus, higher levels of active Cdk1, although a more direct role cannot be excluded.

A sustained activity of Cdk1 was required for the Rif1 phosphorylation and the maintenance of this modification during cell cycle arrest. This could be because the mitotic activity of Cdk1 remains high in budding yeast cells arrested in G2/M.^44–46^ Therefore, the longer the G2/M phase, the longer the time for Cdk1 to phosphorylate even more targets, potentially including Rif1. Alternatively, the effect of CDK on Rif1 phosphorylation could be indirect, by inhibiting a mitotic phosphatase required to dephosphorylate Rif1. The fact that the amino acid sequences incorporating S57 (aka S^57^PTPKR) or S110 (aka. S^110^PVTKS) contain the minimal consensus motif for Cdk1 (S/T-P) supports the idea that Cdk1 may be directly responsible for this modification.

Previous mass spectrometry studies of Rif1 have identified S181 and a couple of serine residues in the C-terminus as showing increased phosphorylation during telomere damage caused by deletion of *YKU70.*^47^ These residues appear to be less important during our WB experiments with Rif1 lacking the C terminus, or with a S181C, Y183F double substitution (Fig. 2G). This could be explained by differences in the sensitivity of the detection methods or by the fact that Rif1 responses to an *yku70Δ* differ from those to a *cdc13-1* mutation.^5^

We have shown that the S57 and S110-dependent phosphorylation of Rif1 had important consequences at the cellular and molecular level. When serine substitutions prevented this Rif1 phosphorylation during telomere damage, cells grew better than those with phosphorylated Rif1 homologues (Fig. 4). Moreover, the serine substituted forms of Rif1 peaked several fold higher on DNA damaged chromosomes and lasted longer than that of nonsubstituted Rif1 (Fig. 5). However, deletion of a larger region around these serines (residues 2–176, aka the PGD) made cells grow poorly and partially stopped Rif1 from accumulating on chromosome during telomere damage (Fig. 2A to C). The latter information could be explained by a nucleus localization signal (NLS) present within the first 176 residues, as previously suggested.^19^

## THE PLIERS MODEL

To explain our findings, we suggest that the PGD at the N-terminus of Rif1 is acting like a “gate” for damaged DNA to access the relevant domains of Rif1, yet this access is perturbed if “the gate” becomes “locked” when phosphate groups (acting as the “key”) are added. This analogy could be expanded into a speculative model we called the “Pliers model” in which two Rif1 dimers assembly together on DNA, to form a pair of molecular pliers.

The Pliers model is based upon previous studies of the crystal structure of the region adjacent to the PGD, called the NTD (residues 177–1283).^19^ The NTD has an elongated crook shape, consistent of a hook, boundary and shaft, as summarized in Fig. 6A.^19^ In the presence of DNA, two NTD monomers assemble themselves head to tail, forming an 8-shaped dimer (Fig. 6B). DNA is threading through the channel of the dimers with help from two loops (Fig. 6C) and it can also bridge adjacent dimers.^19^ According to the Pliers model, the PGD regulates the interaction between one or two Rif1 dimers and damaged DNA. It could be that in its native form, the PGD acts to reduce the gap between two NTD monomers, thus sequestrating the DNA (Fig. 6D). When PGD becomes phosphorylated, this gap widens, allowing the release of DNA (Fig. 6E).

Previous data have also shown that two NTD dimers could be bridged by the same molecule of DNA.^19^ When drawing the bridged dimers, we thought that the structure looked like a pair of pliers with the PGD at its tip, hence the name “Pliers” (Fig. 6F). When PGD is in its native form, the Rif1 pliers assemble onto DNA in a “closed” position, thus facilitating the retention of DNA (Fig. 6F). When PGD is phosphorylated, the Rif1 pliers are opening and DNA is released (Fig. 6G). A sustained activity of Cdk1 is required to keep the Rif1 pliers open and therefore off chromosomes, since phosphorylation of Rif1 is lost soon after the Cdk1 inactivation (Fig. 3D).

Why is Rif1 phosphorylation important during telomere damage? Our data shows that in the absence of this Rif1 phosphorylation, there is an excessive accumulation of Rif1 on DNA damaged chromosomes. As we know from previous work, such accumulation would disrupt the checkpoint and other DNA damage responses (DDR), thus potentially facilitating chromosomal instability due to proliferation of cells with DNA damage. We propose that after an initial association with the DNA damage, Rif1 dissociates from DNA, due to a S57, S110-dependent phosphorylation, so that other, Rif1-independent DDR events, could take place. Conversely, in the absence of its phosphorylation, it may take longer for Rif1 to be removed from the damage (most likely by protein degradation), which may result in prolonged Rif1 effects and delayed Rif1-independent DDR. More work is needed to understand the significance of the S57, S110 dependent Rif1 phosphorylation during other types of damage.

## MATERIALS AND METHODS

### Yeast Strains, Cell Culture, Serial Dilution and Cell Cycle Analysis

All yeast strains were in the W303 background, created either by genetic crossings or by transformation using Longtine plasmids, as described previously.^48^ Targeted mutagenesis of Rif1 was performed by transforming cells with pFA6a-HisMX6 plasmids containing fragments of *RIF1* with relevant nucleotide substitutions generated using GeneArt Gibson assembly cloning method, according to the instructions manual (ThermoFisher Scientific). Cells were incubated in YPD media (Yeast Extract, Peptone, Dextrose) containing 50 mg/l adenine. For experiments analysing the fitness of *cdc13-1* mutant strains at different temperatures, cells grown overnight at 21 °C were diluted to 1.5x10^7^cells/mL, followed by 5-fold dilution series in 96-well plates. Droplets of serially diluted cell culture were then transferred to several YPD plates, with help from sterile metal prongs. Plates were incubated for 3 days at different temperatures. To monitor the cell cycle, cells were stained with DAPI and counted by fluorescent microscopy.

### Chromatin Immunoprecipitation (ChIP)

Chromatin immunoprecipitation (ChIP) was performed as previously described.^49^ Rif1-Myc was immunoprecipitated with mouse monoclonal anti-myc antibodies (Sc-40, Santa Cruz). The immunoprecipitated fraction and the input fraction were quantified by real-time PCR (StepOne Plus, Applied Biosystems) using genomic DNA standards.

### Western Blotting

To detect Rif1 phosphorylation, overnight cultures grown at 21 °C to a concentration of 1 × 10^7^ cells/mL were diluted 1:1 with fresh YPD, and then incubated at 36 °C in a water bath, with shaking. To detect Rif1 the phosphorylation under genotoxic or other type of stress, overnight cultures grown and diluted as above were incubated in the presence of one of the followings: 15 µg/mL nocodazole, 50 µg/mL Phleomycin, 100 mM hydroxyurea, 0.05% w/v methyl-methane sulfonate (MMS). For UV radiation, 30 J/m^2^ were used. The incubation time was 3 h at 23 °C, except for nocodazole, when it was 4 h at 30 °C. For the inhibition of the Cdk1 activity, we incubated *cdc28-as1 cdc13-1*cells in 500 mM NM-1-PP1 at 36 °C, for either 2, 4, or 6 hours. Mock treatment was 0.5% (v/v) DMSO. Proteins were extracted with 10% TCA and resolved on 7% gels. Rif1-Myc, Rif1CΔ-Myc, Rif1-PP1 and Rif1(2–176)Δ were detected with a mouse monoclonal anti-myc antibodies (Sc-40, Santa Cruz). Rad53 protein was detected with rabbit polyclonal anti-Rad53 antibodies (Abcam, ab104232). Secondary antibodies were rabbit anti-mouse (HRP) and goat anti-rabbit (HRP) from Abcam (ab97046 and ab205718, respectively).

## Data Availability

https://doi.org/10.25405/data.ncl.c.6458311
